# Streptozotocin-Induced Hyperglycemia Affects the Pharmacokinetics of Koumine and its Anti-Allodynic Action in a Rat Model of Diabetic Neuropathic Pain

**DOI:** 10.3389/fphar.2021.640318

**Published:** 2021-05-13

**Authors:** Li-Xiang Ye, Hui-Hui Huang, Shui-Hua Zhang, Jing-Shan Lu, Da-Xuan Cao, Dan-Dan Wu, Pei-Wang Chi, Long-Hui Hong, Min-Xia Wu, Ying Xu, Chang-Xi Yu

**Affiliations:** ^1^ Fujian Center for Safety Evaluation of New Drug, Fujian Medical University, Fuzhou, China; ^2^ Department of Pharmacology, College of Pharmacy, Fujian Medical University, Fuzhou, China; ^3^ Fujian Key Laboratory of Natural Medicine Pharmacology, Fujian Medical University, Fuzhou, China; ^4^ Fujian Key Laboratory of Drug Target Discovery and Structural and Functional Research, Fuzhou, China; ^5^ Electron Microscopy Laboratory of Public Technology Service Center, Fujian Medical University, Fuzhou, China

**Keywords:** koumine, diabetic neuropathic pain, pharmacokinetics, pharmacodynamics, anti-allodynic action, streptozotocin-induced hyperglycemia

## Abstract

Koumine (KM), the most abundant alkaloid in *Gelsemium elegans*, has anti-neuropathic, anti-inflammatory, and analgesic activities; thus, it has the potential to be developed as a broad-spectrum analgesic drug. However, factors determining the relationship between analgesic efficacy and the corresponding plasma KM concentration are largely unclear. The pharmacokinetics and pharmacodynamics of KM and their optimization in the context of neuropathic pain have not been reported. We investigated the pharmacokinetics and pharmacodynamics of KM after oral administration in a streptozotocin-induced rat model of diabetic neuropathic pain (DNP) using a population approach. A first-order absorption and elimination pharmacokinetics model best described the plasma KM concentration. This pharmacokinetic model was then linked to a linear pharmacodynamic model with an effect compartment based on the measurement of the mechanical withdrawal threshold. KM was rapidly absorbed (time to maximum plasma concentration: 0.14–0.36 h) with similar values in both DNP and naïve rats, suggesting that DNP did not influence the KM absorption rate. However, the area under the curve (AUC_0–∞_) of KM in DNP rats was over 3-fold higher than that in naïve rats. The systemic clearance rate and volume of KM distribution were significantly lower in DNP rats than in naïve rats. Blood glucose value prior to KM treatment was a significant covariate for the systemic clearance rate of KM and baseline value of the threshold. Our results suggest that streptozotocin-induced hyperglycemia is an independent factor for decreased KM elimination and its anti-allodynic effects in a DNP rat model. To the best of our knowledge, this is the first study to investigate the role of DNP in the pharmacokinetics and pharmacokinetics-pharmacodynamics of KM in streptozotocin-induced diabetic rats.

## Introduction

Diabetic neuropathic pain (DNP) is a chronic pain resulting from a lesion or disease of the nervous system after prolonged hyperglycemia, and is commonly characterized by a burning sensation and tactile allodynia (pain due to a normally non-noxious stimuli) (Gordois et al., 2003; Feldman et al., 2019). Diabetes has been recognized to affect the pharmacokinetics (PK) and pharmacodynamics (PD) of drugs (Dostalek et al., 2012; Qiu et al., 2020). Hyperglycemia causes changes in gene expression, protein glycosylation, epigenetic regulation of PK- or PD-related proteins, and microvascular effects (Thomas et al., 2004; Pirola et al., 2010; Dostalek et al., 2011). Despite the potential effect of diabetes on PK or PD, the role of hyperglycemia in the inter-individual variability of PK and PD parameters is still unclear.

As the most abundant alkaloid in *Gelsemium elegans* Benth., koumine (KM) has been demonstrated to effectively improve the mechanical withdrawal threshold (MWT) in streptozotocin (STZ)-treated rat models and to have a low toxicity. This indicates that KM may be a candidate for the treatment of DNP (Ling et al., 2014). Our pervious study showed that KM-mediated analgesia may involve the activation of translocator protein (18 kDa), inhibition of spinal neuroinflammation, and enhancement of autophagy (Xiong et al., 2017; Jin et al., 2018). Orally administered KM is rapidly absorbed (time to the maximum plasma concentration: 0.23–0.46 h) and has a short half-life (t_1/2_) of 0.78–1.60 h in rats (Wang et al., 2018a; Wang et al., 2018b; Su et al., 2020; Ye et al., 2020). Considering the possibility of drug-disease interactions when KM is used to treat DNP, it is important to understand the relation between DNP relief and the PK and PD of KM. However, the PK and PD of KM and their optimization in the context of neuropathic pain have not been reported.

PK-PD modeling is an important approach to characterize the dynamic relationships between *in vivo* processes and drug efficacy. PK-PD modeling has been widely used in preclinical *in vivo* studies; it contributes to a comprehensive and accurate estimation of dose-response-time data, and thus provides valuable references for understanding and improving drug efficacy, optimizing clinical dosage, reducing toxicity and adverse effects, and identifying clinical implications. In this study, we explored the PK, PD, and population PK-PD correlation of plasma KM concentration and its analgesic activity in a DNP rat model.

## Materials and Methods

### Chemicals and Reagents

STZ was purchased from Sigma-Aldrich (Shanghai, China). KM [purity >99.5%, as determined by high-performance liquid chromatography (HPLC)] and gelsemine (GM) (purity >92.8%, as determined by HPLC) –used as internal standards in PK studies–were isolated from *Gelsemium elegans* according to our previously reported method (Su et al., 2011). HPLC-grade methanol was purchased from Merck (Darmstadt, Germany), and analytical grade formic acid was obtained from Anaqua™ Chemicals Supply (Houston, TX, USA). Deionized water was produced using a Millipore Direct-Q® water purification system (Billerica, MA, USA). KM was dissolved in 0.9% sterile saline (NS) and orally administered to rats at doses (0.28, 1.4, and 7.0 mg kg^−1^ for KM) that previously exhibited anti-allodynic activity in a STZ-induced diabetic model of neuropathic pain (Ling et al., 2014). All other chemicals were of analytical grade.

### Animals

Male Sprague–Dawley (SD) rats (6–8 weeks old; 180–200 g; specific pathogen-free) were obtained from Charles River (Zhejiang, China). The animals, housed in clear plastic cages, were acclimatized for at least 5 days before the experiments. The specific pathogen-free environmental conditions were maintained as follows: 12/12 h light/dark cycle, 40–70% relative humidity, and 20–26°C. The rats were provided free access to water and food *ad libitum*, but fasted overnight before drug administration with free access to water. All experimental procedures and protocols were reviewed and approved by the Animal Ethics Committee of Fujian Medical University (permit number: 2017-01). All experiments in this study were performed with the aim of minimizing animal suffering. All methods and results were reported according to the ARRIVE guidelines (Kilkenny et al., 2010).

### Assessment of MWT

The MWT test protocol was established according to previous studies (Ling et al., 2014). Before the test, SD rats were placed in a transparent organic glass box (approximately 30 × 20 × 20 cm) with a glass plate on top and metal mesh at the bottom and habituated for 30 min before the measurement. Then, the MWT was assessed using an electronic von Frey pain measurement instrument (Model 2391; IITC Life Science, Woodland Hills, CA, United States) using the central plantar surface of the right hind paw. The duration of each stimulation was <1 s, and the stimulation interval was 30 s. The stimulation force (maximum: 55 g) was automatically recorded using the pain meter when the rats shrank, lifted, or licked their paws. The MWT was calculated as the mean of triplicate measurements of minimal force required to observe a response. The mean MWT of triplicate evaluations (once a day for three consecutive days) prior to STZ injection was defined as the baseline MWT.

### Development of STZ-Induced Diabetic Model for Neuropathic Pain

The STZ-induced diabetic rat model was established according to a previous study with minor modifications (Sun et al., 2012; Ling et al., 2014). Briefly, overnight-fasted SD rats (198–235 g body weight) were intraperitoneally injected with a single dose of STZ (70 mg kg^−1^); age-matched naïve rats were administered vehicle (sterile 0.9% NS). Blood samples collected from the tail vein were tested using a portable glucose meter (maximum: 33 mmol L^−1^; One-Touch Ultra, Life Scan, Shanghai, China). Rats with fasting blood glucose levels above 16.6 mmol L^−1^ (300 mg dl^−1^) were considered diabetic and included in the study. Fasting blood glucose level and body weight were checked every week for 21 days after STZ injection. The fasting blood glucose level prior to STZ injection was defined as the baseline blood glucose. Twenty one days after intraperitoneal STZ administration, 42 diabetic rats with a >20% decline in MWT compared with the baseline value were classified as rats with DNP, of which 24 rats were used in the PD study and 18 rats were used in the PK study on day 22.

### PD Studies

Twenty-four STZ-induced DNP rats were evenly assigned to four groups: the vehicle-treated group (vehicle), and low-dose, medium-dose, and high-dose KM groups. Six aged-matched naïve SD rats comprised the naïve group (naïve) and received oral gavage of NS. Rats in the low-dose, medium-dose, and high-dose KM groups received 0.28, 1.4, and 7.0 mg kg^−1^ KM, respectively, by oral gavage. The MWT was measured at 0, 0.083, 0.167, 0.333, 0.667, 1, 2, 4, 6, 8, and 12 h after administration. Curves of the threshold for paw withdrawal over time were plotted. Thereafter, the area under the curve (AUC) of MWT against time was calculated.

### PK Studies

Eighteen STZ-induced DNP rats were randomly assigned to three groups (*n* = 6 per group); they received a single dose of 0.28, 1.4, or 7.0 mg kg^−1^ KM by oral gavage. Eighteen body-weight-matched naïve SD rats were used as controls and randomly assigned to three groups (*n* = 6 per group) and received a single dose of 0.28, 1.4, or 7.0 mg kg^−1^ KM by oral gavage. The rats were anesthetized with 3% isoflurane (RWD Life Science, Shenzhen, China) before blood collection. Approximately 0.3 ml of blood samples was collected from the postorbital venous plexus of anesthetized rats in heparinized tubes at 0.083, 0.167, 0.333, 0.667, 1, 2, 4, 6, 8, and 12 h after the administration of KM. The samples were immediately centrifuged at 2,000 × *g* for 5 min to obtain plasma, which was stored at −80°C until further analysis.

### KM Plasma Concentration Analysis

The preparation and quantification of KM in the plasma were performed as previously reported (Ye et al., 2020). In brief, 50 μL of plasma was spiked with GM at a final concentration of 80 ng ml^−1^ and evaporated to dryness. The dry sample was mixed with 400 μL of ethyl acetate by vortexing and then centrifuged at 12,000 × *g* for 10 min at 4°C. The supernatant was carefully transferred into fresh tubes and evaporated to dryness. The residues were redissolved with 100 μL of 50% methanol solution, of which 5 μL was injected for ultra-performance liquid chromatography (UPLC)–tandem mass spectrometry (MS/MS) analysis. The assay was performed on an Agilent 1290 Infinity UPLC^®^ system (Agilent Technologies, Santa Clara, CA, United States). The mobile phase comprised methanol and water with 0.1% formic acid. The flow rate was 0.4 ml min^−1^ at 40°C. The gradient program is shown in SupplementaryTable S1. Only the eluent between 2.0 and 5.0 min was passed into the MS/MS system, operated in the positive ionization mode. The multiple reaction monitoring transition of KM was at m/z 307.2/180.1 and GM was at m/z 323.1/70.1.

### Non-Compartmental PK Analysis

PK data were first estimated using a non-compartmental analysis with Phoenix® WinNonLin™ 8.2 software (Pharsight Corporation, Certara, CA, United States). The time (T_max_) to maximum observed plasma concentration was determined directly from the observed values. The AUC from time zero to infinity (AUC_0-∞_) was calculated using the log-linear trapezoidal method. The t_1/2_ was calculated as ln (2)/(slope of the terminal log-linear phase). Systemic clearance (CL) was calculated as dose/AUC_0-∞_. The apparent volume of distribution (V_d_) was determined as CL/(slope of the terminal log-linear phase) (Kim and Baek, 2018; Ye et al., 2020).

### Population PK-PD Model Development

#### Data Source, Software, and Criteria

The data of population PK analyses of plasma KM concentrations and PD analyses of the KM response in the MWT test were pooled and analyzed. However, the PK parameters were separated from the PD parameters because the PK and PD data were obtained from separate experiments. The population modeling approach can combine these data and model them simultaneously via rigorous statistical methods (Mould and Upton, 2012; Mould and Upton, 2013; Upton and Mould, 2014).

Population PK and PD analyses were performed using Phoenix® NLME^™^ 8.2 software (Pharsight Corporation, Certara, CA, United States). The PK-PD model was developed using a non-linear mixed effects (NLME) modeling approach with first-order conditional estimation (FOCE)-extended least squares analyses. The PK model development and parameter estimation were performed separately from the PD model, and the best PK model was fixed when developing the PD models.

The development of PK and PD models was based mainly on the minimum value of twice the negative log likelihood (-2LL) provided by NLME. A decrease in -2LL of at least 6.63 units was considered as a significant improvement of the model (χ^2^, *p* < 0.01, df = 1) (Mould and Upton, 2013). The bias in model fits was detected by visual inspection of the classic goodness-of-fit plots, including observations (OBS) vs. individual predictions (IPRED) or population predictions (PRED); conditional weighted residuals (CWRES) vs. independent variables (IVAR), PRED, or time; the coefficient of variation (%CV) < 50% in parameter estimates; and the examination of shrinkage <40% (Karlsson and Savic, 2007).

#### General Model Building Process

There are usually two sources of variability in a population analysis: between-subject variability (BSV) and residual variability (RV). It is generally assumed that the BSV of the parameters has a log-normal distribution based on the exponential relationship. Therefore, the model parameter for the *i* ^*th*^ subject (*θ*_*i*_) is presented as

$$\theta_{i}=\theta_{tv}\times Exp\left(\eta_{i}\right),$$ (1)

where *θ*_*i*_ is the parameter estimate for the *i* ^*th*^ subject and *θ*_*tv*_ is the typical value of parameter *θ* for the population. *η*_*i*_, a random effect of BSV, denotes the deviation between *θ*_*i*_ and *θ*_*tv*_, which follows a normal distribution with mean zero and variance *ω* ^2^.

For RV, a log-additive residual error model was examined, as described below:

$$\ln\left(C_{ij}\right)=\ln\left({\hat{C}}_{ij}\right)+\varepsilon_{ij},$$ (2)

where *C*_*ij*_ and ${\hat{C}}_{ij}$ are the observed and predicted KM concentrations in the plasma at the *j* ^*th*^ time point in the *i* ^*th*^ subject, respectively. *ε*_*ij*_ is a random effect of RV generally assumed to be normally distributed with mean zero and variance σ^2^.

We also attempted to establish the initial model using diagonal or non-diagonal covariance matrices. Model parameters together with their relative standard error (RSE) are presented as the estimates. The degree of BSV or RV is expressed as the %BSV or %CV. When a normal distribution of parameters is assumed, the following equation converts the variance to %BSV/%CV in the original scale.

$$\%BSV\;or\;\%CV=\;\sqrt{Exp\left(\omega^{2}\;or\;\sigma^{2}\right)-1}\times 100,$$ (3)

A visual predictive check (VPC) and a nonparametric bootstrap (NPB) were applied as internal evaluation methods to qualify the robustness and predictive performance using Phoenix® NLME^™^ 8.2 software (Pharsight Corporation, Certara, CA, United States). For the VPC, simulations (*n* = 500) were performed using the final population model parameters. The observed data and their 5^th^, 50^th^, and 95^th^ simulated percentiles were used to assess model performance (Yano et al., 2001). The agreement between the observed values and model-based simulations was judged visually. An NPB procedure (*n* = 500) with resampling technique was utilized to obtain the mean, median and 95% confidence intervals (2.5^th^ and 97.5^th^ percentiles) for the model parameters, and then compared with the model parameters estimated from the original observations (Parke et al., 1999).

#### Population PK Structure

Population PK models were structured on apparent V_d_ and CL. We considered different compartment models with first-order or zero-order elimination to determine the best fit for the KM concentration data. The final PK model was developed by evaluating the effects of subject-specific covariates: dosage, body weight, blood glucose prior to KM treatment, and MWT prior to KM treatment. The first covariate was modeled as categorical, and last three covariates were modeled as continuous. These covariates were first examined using η vs. candidate covariates using box plots or scatter plots, and then added to and excluded from the population model in a stepwise manner. In the forward selection process, a covariate contributing to more than 6.63-fold reduction in the objective function value (*α* = 0.01) was considered significant; and in the backward elimination process, a covariate excluded from the model contributing to more than 10.8-fold increase in the objective function value was considered significant (*α* = 0.001) (Wählby et al., 2002; Zhang et al., 2020).

If a continuous covariate was added to the population model, Eq. 4 was modified to show its effect on the model parameter as described below:

$${\theta^{\prime }}_{i}=\theta_{tv}\times {\left(\frac{Cov}{Med_{Cov}}\right)}^{\theta_{Cov}}\times Exp\left(\eta_{i}\right),$$ (4)

where Cov is the value of the continuous covariate that affects parameter *θ* and Med_Co*v*_ is the median of Cov. *θ*_cov_ is the parameter estimate for Cov*.*

If the categorical covariate dosage was added to the population model, Eq. 5 was modified to show its effect on the model parameter as described below:

$${\theta^{\prime }}_{i}=\theta_{tv}\times \prod_{k=1}^{3}Exp\left(\theta_{Dose,k}\times Dose_{k}\right)\times Exp\left(\eta_{i}\right),$$ (5)

where *θ*_Dose*,k*_ is the parameter estimate for the *k* ^*th*^ dosage (*k* = 1, 2, or 3). When *the k* ^*th*^ dosage was administered, the Dose_*k*_ was 1, the dose in others was 0. When the dose was 0, *θ*_*i*_^*’*^ was equal *to θ*_*i*_.

#### Population PK and PD Modeling for KM Treatment

The PD model was sequentially structured based on the fixed PK model. For PD, we examined the linear, effect compartment, sigmoidal maximum effect constant (E_max_), and indirect effect models. For the indirect effect model, we tested stimulation inverse, stimulation infinite, inhibition limited, inhibition inverse build up, and loss models as well as a sigmoidal model incorporating a Hill factor fixed at 1, and examined the best residual model similar to the PK model. In the covariate modeling, the effects of dosage, body weight, blood glucose prior to KM treatment, and MWT prior to KM treatment were evaluated by screening, followed by a stepwise process similar to that used for PK modeling.

### Statistics

The data are presented as mean ± standard error of the mean (SEM) unless otherwise stated, and plotted and analyzed using Prism 8.0 (GraphPad Software, San Diego, CA, USA). All normally distributed data were statistically analyzed with parametric tests, including Student’s *t*-test and one-way or two-way analysis of variance (analysis of variance, repeated measures) followed by Tukey–Sidak *post hoc* analysis. All non-normally distributed data were analyzed with nonparametric statistics, including Friedman analysis of variance test, followed by Mann–Whitney *U* test for *post hoc* comparison. Unless otherwise specified, the significance level was set at *p* < 0.05.

## Results

### STZ-Induced Diabetic Rats had Hyperglycemia, Tactile Allodynia, and Weight Loss Compared with Naïve Rats

**FIGURE 1:**
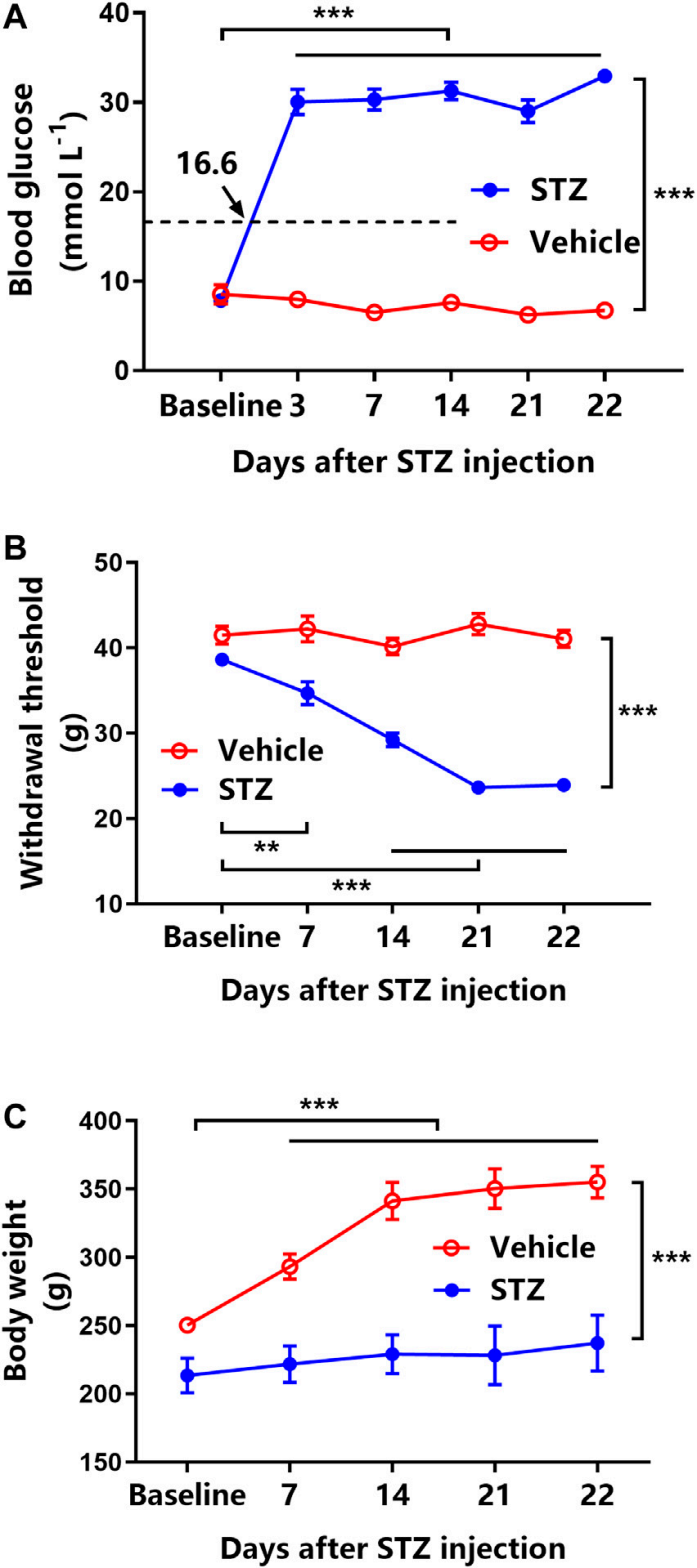
Streptozotocin (STZ) induces hyperglycemia, tactile allodynia, and body weight loss in rats (*n* = 6 for vehicle-treated rats, *n* = 42 for STZ-induced diabetic rats). **(A)** Time course of blood glucose levels in vehicle-treated rats (open circles) and STZ-induced rats (filled circles). ***represents *p* < 0.001 between the baseline and experimental values. **(B)** Mechanical withdrawal threshold (MWT) of vehicle-treated rats (open circles) and STZ-induced diabetic rats (filled circles). **represents *p* < 0.01 and ***represents *p* < 0.001 between the baseline and experimental values. **(C)** The changes in body weight in vehicle-treated rats (open circles) and STZ-induced diabetic rats (filled circles). ***represents *p* < 0.001 between the baseline and experimental values.

Intraperitoneally injected STZ considerably elevated the blood glucose level (all >16.6 mmol L^−1^) from 3 to 22 days after administration (*p* < 0.001, Figure 1A). STZ-induced diabetic rats exhibited a >20% reduction in MWT starting from day 7 and further decreased till day 21 after STZ injection (*p* < 0.001, Figure 1B). Furthermore, compared with naïve rats, STZ-induced diabetic rats exhibited a significant loss of body weight from 7 to 22 days (*p* < 0.001, Figure 1C). However, vehicle-treated rats maintained their baseline blood glucose level and MWT along with approximate 100 g body weight gain during the 22 days (Figures 1A–C).

### KM Exhibited a Dose-Dependent Anti-Allodynic Effect After a Single Oral Dose in STZ-Induced DNP Rats

**FIGURE 2:**
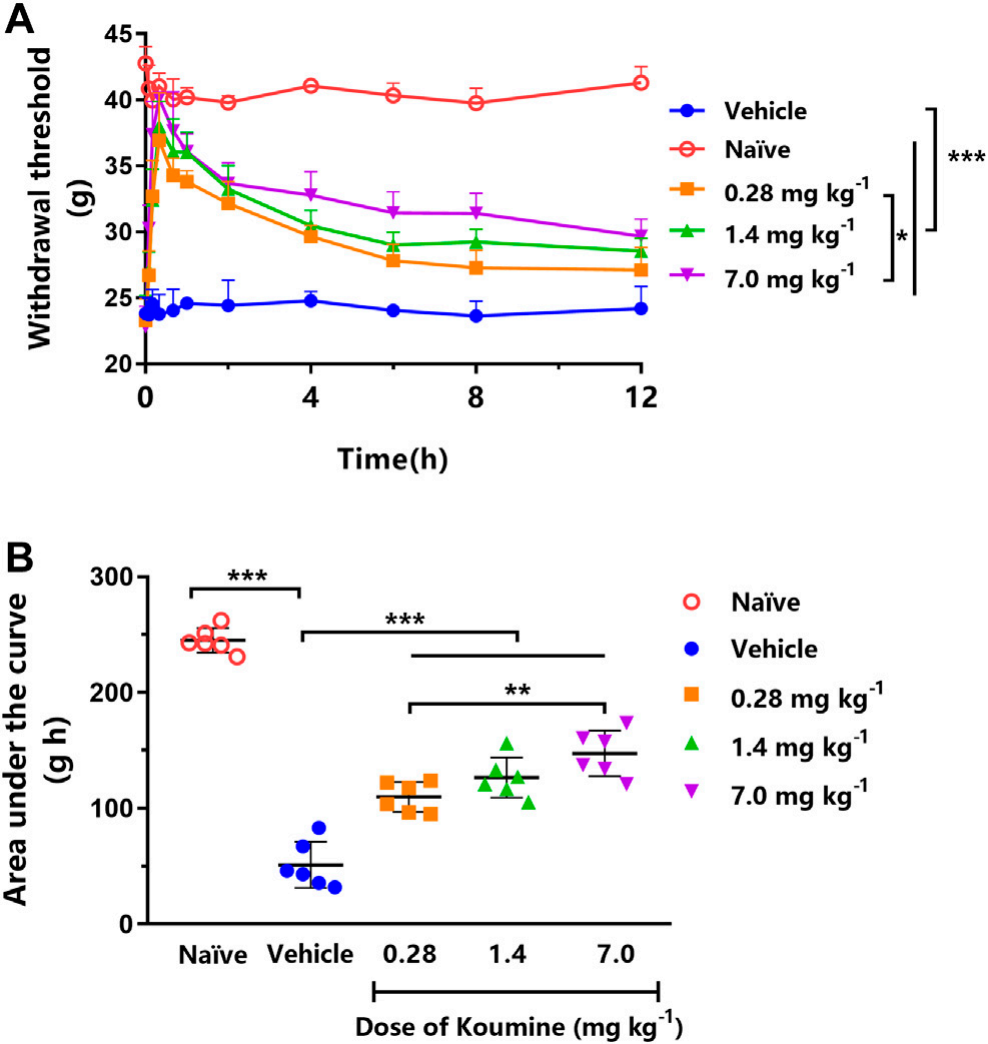
Effect of koumine (KM) on mechanical allodynia in rats with streptozotocin (STZ)-induced diabetic neuropathic pain. **(A)** Dose-dependent anti-allodynic effect of KM following oral gavage in vehicle-treated STZ-induced diabetic neuropathic pain rats (filled circles) and those treated with 0.28 mg kg^−1^ (squares), 1.4 mg kg^−1^ (triangles), and 7.0 mg kg^−1^ (inverted triangles) KM. **(B)** The area under the curve (AUC) of the data from graph A. ^*^ represents *p* < 0.05, **represents *p* < 0.01, and ***represents *p* < 0.001 among different treatments (*n* = 6 per group).

Each dose of orally administered KM (0.28, 1.4, and 7.0 mg kg^−1^) significantly increased the MWT compared with the vehicle treatment in STZ-induced DNP rats (all *p* < 0.001, Figure 2A). Furthermore, the effect of 7.0 mg kg^−1^ KM was significantly stronger than that of KM at 0.28 mg kg^−1^ (*p* < 0.05, Figure 2A). The AUCs for MWT after the oral administration of KM (0.28, 1.4, and 7.0 mg kg^−1^) were significantly higher than after vehicle treatment in STZ-induced DNP rats (all *p* < 0.001, Figure 2B). The AUC for MWT after 7.0 mg kg^−1^ KM administration was 1.34- and 2.88-fold higher than that after 0.28 mg kg^−1^ KM and vehicle administration, respectively (147.4 ± 8.0 vs. 109.8 ± 5.3 and 51.2 ± 8.1, respectively).

### Validation of UPLC-MS/MS for KM Detection in the Rat Model of STZ-Induced DNP

The full-scan precursor ions and products of KM and GM are shown in Supplementary Figures S1A,B. The representative chromatograms presented in Supplementary Figures S1C–F are for blank rat plasma, blank rat plasma spiked with KM (final concentration: 20.0 ng ml^−1^) and GM (final concentration: 80 ng ml^−1^), naïve rat plasma collected 0.17 h after a single oral administration of 7.0 mg kg^−1^ KM and spiked with GM, and STZ-induced DNP rat plasma collected 0.17 h after a single oral administration of 7.0 mg kg^−1^ KM and spiked with GM. The calibration curves for KM (0.2–200 ng ml^−1^) were fitted to a 1/*x* ^2^ weighted least squares linear regression model. The intra-day and inter-day precisions for the four levels of quality control (QC) samples (lower limit of quantitation [LLOQ], low, medium, and high) ranged from 1.6 to 12.7% and 3.5% to 13.8%, respectively (see detailed method validation data in Supplementary Table S2). At the LLOQ of 0.2 ng mL^−1^ KM, the average inter-day deviations of the predicted concentrations from the nominal values were within 2%. The matrix effects for the QC samples were 100.0–108.0% for KM (%CV: < 6%). The mean extraction recoveries of KM ranged from 59.0 to 67.2% among QC samples (Supplementary Table S3). The detailed stability KM data are shown in Supplementary Table S4.

### PK Profiles of Single-Dose Orally Administered KM in DNP

**FIGURE 3:**
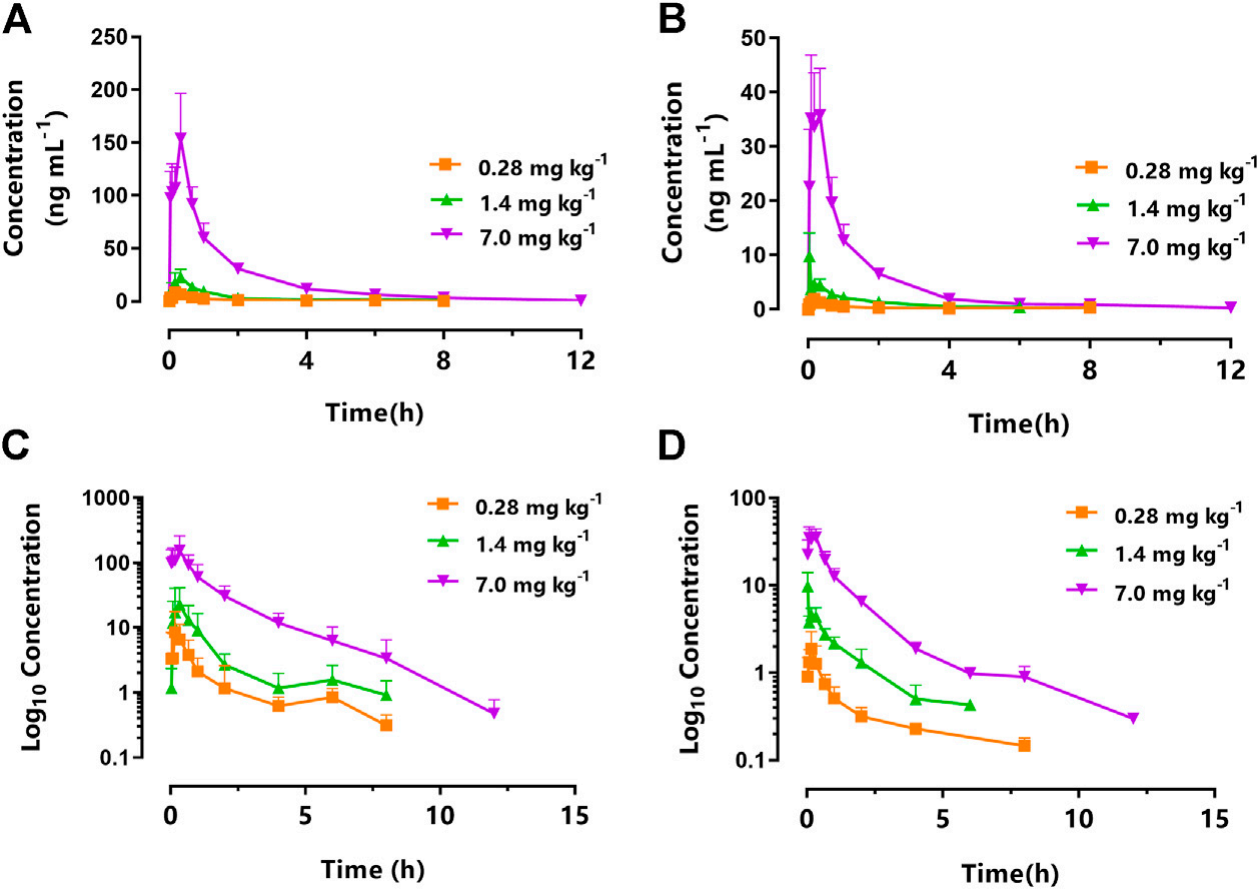
Mean plasma concentration-time curve of koumine (KM) following intragastric administration of 0.28 mg kg^−1^ (squares), 1.4 mg kg^−1^ (triangles), and 7.0 mg kg^−1^ (inverted triangles) to **(A)** rats with streptozotocin (STZ)-induced diabetic neuropathic pain or **(B)** naïve rats. The corresponding log_10_ concentration-time curve of koumine (KM) following intragastric administration of 0.28 mg kg^−1^ (squares), 1.4 mg kg^−1^ (triangles), and 7.0 mg kg^−1^ (inverted triangles) to **(C)** rats with streptozotocin (STZ)-induced diabetic neuropathic pain or **(D)** naïve rats (*n* = 6 per group).

Figure 3 displays the mean plasma concentration-time profiles on a linear (Figure 3A) or log10-linear (Figure 3C) scales of KM (0.28, 1.4, and 7.0 mg kg^−1^) after oral gavage in STZ-induced DNP rats. The PK parameters calculated using the non-compartmental model are summarized in Table 1. In accordance with our previously published studies (Ye et al., 2020), the absorption of KM in the gastrointestinal tract was rapid (T_max_: 0.21–0.36 h) for all doses in DNP rats. KM was detected at 0.083 h in the majority of DNP rats. The AUC_0–∞_ of the 7.0 mg kg^−1^ dose was over 27-fold higher than that of the 0.28 mg kg^−1^ dose (220.66 ± 23.64 vs. 8.10 ± 2.58 ng h ml^−1^; *p* < 0.001; Table 1) and nearly 8-fold higher than that of the 1.4 mg kg^−1^ dose (220.66 ± 23.64 vs. 27.69 ± 6.68 ng h ml^−1^; *p* < 0.001; Table 1). The t_1/2_ of the 7.0 mg kg^−1^ dose was 3.6-fold longer than that of the 0.28 mg kg^−1^ KM dose in DNP rats (3.55 ± 1.36 vs. 0.97 ± 0.35 h; *p* < 0.01; Table 1) and over 2-fold longer than that of the 1.4 mg kg^−1^ dose (3.55 ± 1.36 vs. 1.63 ± 0.44 h; *p* < 0.05; Table 1). The V_d_ of KM was significantly lower for the 0.28 mg kg^−1^ dose than for the 7.0 mg kg^−1^ dose (*p* < 0.01; Table 1). There was a slight decrease in CL for the 7.0 mg kg^−1^ dose, but this did not significantly differ among the three doses (*p* > 0.05).

**TABLE 1:** Non-compartmental PK parameters after the oral administration of KM (0.28, 1.4, or 7.0 mg kg^−1^) in naïve and STZ-induced DNP rats (*n* = 6).

Parameters^a^	Administered dose					
	0.28 mg kg^−1^		1.4 mg kg^−1^		7.0 mg kg^−1^	
	Naïve	DNP	Naïve	DNP	Naïve	DNP
T_max_ (h)	0.34 ± 0.11	0.22 ± 0.04	0.14 ± 0.06	0.36 ± 0.07	0.21 ± 0.06	0.21 ± 0.06
AUC_0-∞_ (ng h ml^−1^)	2.46 ± 0.85	8.10 ± 2.58^†††^	7.35 ± 1.72	27.69 ± 6.68^†††^	49.49 ± 8.53***	220.66 ± 23.64***,^†††^
t_1/2_ (h)	0.82 ± 0.19	0.97 ± 0.35	1.10 ± 0.27	1.63 ± 0.44	2.46 ± 0.48**	3.55 ± 1.36**
V_d_ (L kg^−1^)	189.45 ± 51.77	70.36 ± 21.45^†††^	304.92 ± 20.53	150.91 ± 53.53^†††^	551.28 ± 110.56**,^,†††^	182.06 ± 76.86**,^,†††^
CL (L h^−1^ kg^−1^)	215.07 ± 89.02	68.49 ± 24.04^†††^	248.24 ± 54.02	66.40 ± 13.62^†††^	157.81 ± 19.74	33.41 ± 3.21^†††^

a T_max_, time to reach the maximum observed plasma concentration; AUC_0-∞_, area under the curve from time zero to infinity; t_1/2_, half-life; V_d_, apparent volume of distribution; CL, clearance rate.

b * represents p < 0.05, **represents p < 0.01, and ***represents p < 0.001 compared with 0.28 mg kg^−1^ or 1.4 mg kg^−1^ KM; ^†^represents p < 0.05, ^††^represents p < 0.01, and ^†††^represents p < 0.001 compared DNP rats with naïve rats in the same administered dose.

### DNP Significantly Affected the PK Profiles of Rats that Received Single-Dose Orally Administered KM Compared with those of Naïve Rats

The PK parameters of orally administered KM in naïve and DNP rats are summarized in Table 1. Figure 3 displays the mean plasma concentration-time curves on a linear or log10-linear scales of KM (0.28, 1.4, and 7.0 mg kg^−1^) after oral administration to DNP rats (Figures 3A,C) or naïve (Figures 3B,D). Similarly, KM was rapidly absorbed in both naïve and DNP rats. The T_max_ of KM in naïve and DNP rats ranged from 0.14 to 0.36 h (*p* > 0.05). The AUC_0–∞_ for KM in DNP rats was > 3-fold higher than that in naïve rats (220.66 ± 23.64 vs. 49.49 ± 8.53 ng h ml^−1^ for 7.0 mg kg^−1^; 27.69 ± 6.68 vs. 7.35 ± 1.72 ng h ml^−1^ for 1.4 mg kg^−1^; and 8.10 ± 2.58 vs. 2.46 ± 0.85 ng h ml^−1^ for 0.28 mg kg^−1^; *p* < 0.001; Table 1). The t_1/2_ was comparable in the naïve and DNP groups. The CL of KM was significantly lower (by 21.2% for 7.0 mg kg^−1^, 26.7% for 1.4 mg kg^−1^, and 31.8% for 0.28 mg kg^−1^, respectively) in DNP rats than in naïve rats (*p* < 0.001; Table 1). The V_d_ values of KM were significantly lower (by 33.0% for 7.0 mg kg^−1^, 49.5% for 1.4 mg kg^−1^, and 37.1% for 0.28 mg kg^−1^, respectively) in DNP rats than in naïve rats (*p* < 0.001; Table 1).

### Hyperglycemia was an Independent Factor Affecting Single-Dose Orally Administered KM via Population PK Analysis

**FIGURE 4:**
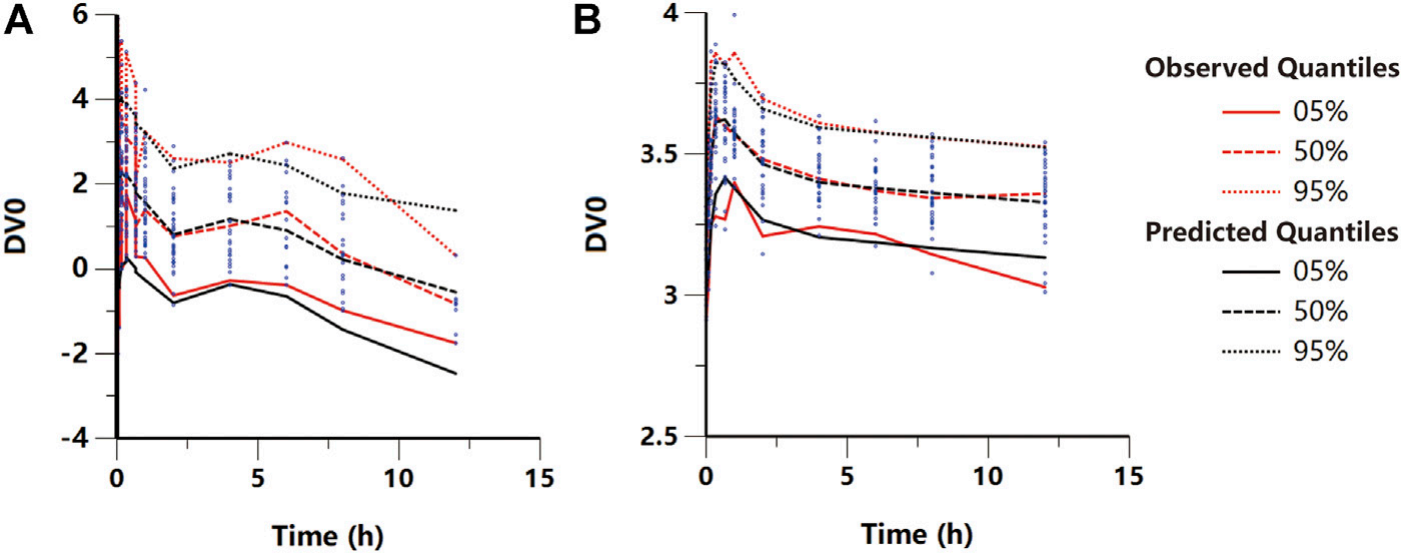
Visual predictive check plot for the final **(A)** pharmacokinetic and **(B)** pharmacodynamic models. Red lines represent the 5^th^, 50th, and 95th percentiles of the observed data around the simulated percentiles (black lines) using the developed model.

A two-compartment model with first-order absorption and elimination best fits the plasma KM PK data as described by the differential Eqs. 6–8 (Supplementary Table S5):

$$\frac{dX_{a}}{dt}=-K_{a}\times X_{a},$$ (6)

$$V_{1}\frac{dC_{1}\left(t\right)}{dt}=K_{a}\times X_{a}+CL_{2}\times C_{2}\left(t\right)-CL_{2}\times C_{1}\left(t\right)-CL_{1}\times C_{1}\left(t\right),$$ (7)

$$V_{2}\frac{dC_{2}\left(t\right)}{dt}=CL_{2}\times C_{1}\left(t\right)-CL_{2}\times C_{2}\left(t\right),$$ (8)

where *X*_*a*_ is the amount of KM at the administered compartment, *K*_*a*_ is the absorption rate constant, *C*_*1*_ and *C*_*2*_ are the KM concentrations in the central and peripheral compartments, respectively; *V*_*1*_ and *V*_*2*_ are the central and peripheral volumes of distribution, respectively; *CL*_*1*_ is the systemic clearance rate, and *CL*_*2*_ is the distribution clearance between the central and peripheral compartments. Among several residual models described in the method, the log-additive error model was found to best fit the residuals and make selections. The non-diagonal covariance matrix was used to establish the final PK model. The covariate blood glucose level prior to KM treatment (*Glu*) and dosage were included to obtain an acceptable goodness-of-fitting result, which affected the *CL*_*1*_ and *V*_*1*_ incorporated as a median-normalized covariates (*Δ*-2LL = −65 points) and well explained the BSV for these parameters. Figure 4A and Supplementary Figure S2 show the results of the VPC and the goodness-of-fit plots of the final population PK model, respectively. These plots suggested that the population PK model accurately described the IPRED and PRED of the KM plasma concentration. The final PK parameter estimates and the results of the bootstrap validation are presented in Table 2. The absolute value of the %RSE of each PK fixed-effect parameter estimate was <30%. The BSV could be estimated for all PK parameters, including the *K*_*a*_, *V*_1_, *V*_2_, *CL*_1_, and *CL*_2_, with respect to the values of shrinkage estimates <40%. The %BSV for each BSV parameter was large (85.1–443.5%). However, the robustness of the population PK model was validated by bootstrap validation. Each parameter estimate obtained from the bootstrap procedure was similar to those obtained from the original dataset with a RSE <10%, indicating that the model adequately estimated the PK model parameters.

**TABLE 2:** Population PK and PD parameters calculated by fitting the model to the observed data after the oral administration of KM (0.28, 1.4, or 7.0 mg kg^−1^) along with bootstrap validation.

Parameters	Unit	Final model		Bootstrap (*n* = 500)		
		Estimate	%RSE	Mean	Median	2.5^th^–97.5^th^ percentiles
Fixed-effect parameters, θ						
*K*_*a*_	h^−1^	11.4	15.7	12.0	11.7	5.1–25.9
*V*_1_	mg mL (kg ng)^−1^	0.075	15.8	0.073	0.072	0.045–0.10
Effect of 1.4 mg kg^−1^ on *V*_1_		1.31	16.8	1.20	1.23	0.30–1.83
Effect of 7.0 mg kg^−1^ on *V*_1_		1.42	20.2	1.42	1.39	0.77–2.03
*V*_2_	mg mL (kg ng)^−1^	0.11	24.1	0.12	0.11	0.063–0.21
*CL*_1_	mg mL (kg ng h)^−1^	0.068	12.7	0.065	0.064	0.045–0.090
*Glu* Effect on *CL*_1_		−0.20	−15.1	−0.21	−0.19	−0.66–0.082
*CL*_2_	mg mL (kg ng h)^−1^	0.052	27.6	0.053	0.051	0.028–0.090
*E*_0_	g	25.1	1.6	25.0	25.0	23.8–25.9
*Glu* Effect on *E*_*0*_		−0.33	−3.5	−0.33	−0.33	−0.37–(-0.31)
*K*_eff_	—	6.2	10.0	6.4	6.3	5.38–8.30
Effect of 1.4 mg kg^−1^ on *K*_eff_		-0.76	-12.0	-0.82	-0.76	−1.37–(−0.34)
Effect of 7.0 mg kg^−1^ on *K*_eff_		−2.1	−9.6	-2.1	-2.2	−2.6–(−1.6)
*K*_*e*_	h^−1^	10.5	28.5	11.8	10.4	6.4–25.0
Between-subject variability, ω						
*K*_*a*_	%	443.5	19.1	—	—	—
*V*_1_	%	96.9	18.2	—	—	—
*V*_2_	%	258.8	3.0	—	—	—
*CL*_1_	%	85.1	12.6	—	—	—
*CL*_2_	%	265.5	1.6	—	—	—
Residual variability, σ						
Log-additive residual error (C)	%	0.50	6.7	0.50	0.50	0.43–0.56
Log-additive residual error (E)	%	0.11	5.1	0.11	0.11	0.10–0.11

Ka, absorption rate constant; C1, KM concentration in the central compartment; C2, KM concentration in the peripheral compartment; V1, central volume of KM distribution; V2, peripheral volume of KM distribution; CL1, systemic clearance rate; CL2, inter-compartmental clearance rate; E, the final effects of KM; E0, the baseline value of the MWT; Keff, slope of linear function in KM effect; Ke, first-order rate constant governing the equilibrium distribution of KM between plasma and the effect compartment; ω: variance of BSV; σ: variance of RV.

### STZ-Induced Hyperglycemia was an Independent Factor Affecting Single-Dose Orally Administered KM via Population PK-PD Analysis

**FIGURE 5:**
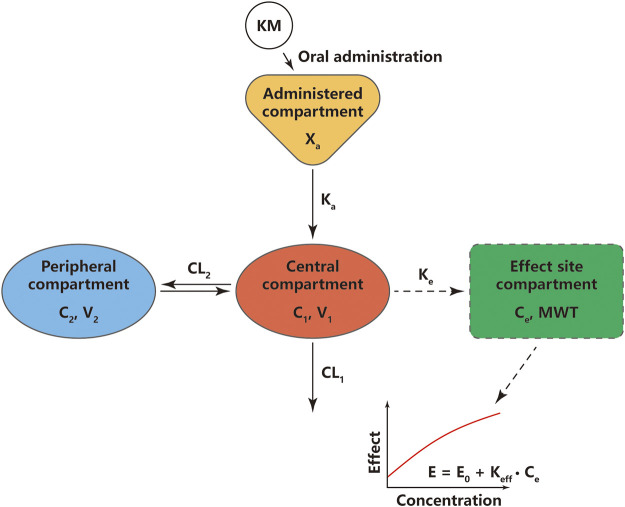
Schematic diagram of pharmacokinetic and pharmacodynamic modeling of koumine (KM) for its effects on mechanical withdrawal threshold (MWT). *X*_*a*_, the amount of KM at the administered compartment; *K*_*a*_, absorption rate constant; *C*_1_, KM concentration in the central compartment; *C*_*2*_, KM concentration in the peripheral compartment; *V*_1_, central volume of KM distribution; *V*_2_, peripheral volume of KM distribution; *CL*_1_, systemic clearance rate; *CL*_2_, inter-compartmental clearance rate; *E*, the final effects of KM; *E*_*0*_, the baseline value of the MWT; *K*_eff_, slope of linear function in KM effect; *K*_*e*_, first-order rate constant governing the equilibrium distribution of KM between plasma and the effect compartment.

A linear model with an effect compartment best described the KM PD data as described by differential Eqs 9, 10:

$$\frac{\text{d}C_{e}}{\text{d}t}=K_{e}\times \left[C_{1}\left(t\right)-C_{e}\left(t\right)\right],$$ (9)

$$\frac{\text{d}E}{\text{d}t}=E_{0}+K_{\text{eff}}\times C_{e}\left(t\right),$$ (10)

where, *C*_*1*_ and *C*_*e*_ are the KM concentrations in the central and effect site compartments, respectively, *E*_*0*_ is the baseline of the MWT, and *K*_eff_ is the slope of the KM effect on MWT. The final graphical PK-PD models are shown in Figure 5, and describe the complete time course of KM and MWT changes. Figure 4B presents the results of the VPC for the final population PK-PD model for MWT changes. The final PD parameter estimates for the MWT and the results of the bootstrap validation are presented in Table 2. The %RSE of each fixed-effect parameter estimate and BSV parameter was acceptable. In several residual models described in the method, the log-additive error model was found to best describe the residuals and make selections. In a stepwise method, the covariate blood glucose level prior to KM treatment (*Glu*) and dosage were included to obtain a better-fitting result, which affected the *E*_*0*_ and *K*_eff_, respectively (*Δ*-2LL = −148 points); this completely explains the BSV for this parameter. Figure 4B and Supplementary Figure S3 show the results of the VPC and goodness-of-fit plots of the final population PK-PD model, respectively. These plots show that the population PK-PD model adequately describes the IPRED and PRED of the KM plasma concentration and KM effect on MWT. The final PD parameter estimates and results of the bootstrap validation are shown in Table 2. Neither PD parameter could be estimated from BSV. The robustness of the population PD model was evaluated by bootstrap validation. Parameter estimates obtained from the bootstrap validation procedure was similar to those obtained from the original dataset with a RSE < 13%, suggesting that the model adequately estimated the PD model parameters.

## Discussion

To the best of our knowledge, this is the first study to investigate the role of DNP on KM PK and PK-PD in STZ-induced DNP rats. Considering the potentially decreased expression and function of enzymes and transporters in experimentally induced diabetic rats, the study was designed with a single oral dose administration. A population PK-PD model was further developed to investigate the influence of STZ-induced hyperglycemia and other potential covariates on KM PK and PD parameters. Our results show that the systemic clearance rate was affected by hyperglycemia.

STZ-induced hyperglycemia, tactile allodynia, and weight loss persisted for more than 7 days without any signs of remission. Our findings are consistent with those of previous studies (Calcutt et al., 1996; Ling et al., 2014; Hernández-Reyes et al., 2019). We report that the oral administration of KM induced a dose-dependent anti-allodynic effect in STZ-treated rats. Moreover, these results are consistent with those of previous studies from our laboratory that subcutaneous injection of KM reduces tactile allodynia in STZ-treated rats (Ling et al., 2014). Using the MWT test as a PD model for DNP, we found that the maximum efficacy of KM was at a dose of 7.0 mg kg^−1^. The minimum effective dose of KM for the DNP model was only 1/5 of that used for other neuropathic pain models, including chronic constriction injury and spinal nerve ligation (Ling et al., 2014). However, the mechanism of the pharmacologic action of KM in DNP is still unknown. Our previous study found that KM might inhibit spinal neuroinflammation, enhance autophagy, and activate translocator protein (18 kDa) in neuropathic pain (Xiong et al., 2017; Jin et al., 2018).

Recently, we developed a sensitive UPLC-MS/MS method that requires only 50 μL of plasma to determine KM concentration, which was successfully utilized in a PK study in aged rats (Ye et al., 2020). The results of method validation presented in Supplementary Figure S1 and Supplementary Tables S2–S4 were consistent with those of our previous study, suggesting that the concentrations of KM detected in the plasma are reliable. According to the findings of this study, the DNP status changes the PK parameters of KM after oral administration. KM was rapidly absorbed with similar T_max_ in naïve and DNP rats, which suggested that the DNP status did not influence the absorption rate of KM. However, the AUC_0–∞_ of KM in DNP rats was >3-fold higher than that in naïve rats (Table 1), and the CL and V_d_ of KM were significantly lower in DNP rats than in naïve rats (Table 1). The altered KM PD in rats with high blood glucose levels could be attributed to STZ-induced hyperglycemia or the factors associated with DNP (threshold, weight, or dosage). These findings raised the question of whether high blood sugar or a low threshold affected the PK of KM.

To further investigate this problem, we conducted a population PK and PD analysis. Population PK and PD modeling was considered a more appropriate approach in this study, as it accounts for individual observations and can assess whether certain covariates can influence PK or PD parameters. The major advantage is that the population modeling method allows the determination of whether individual data can explain differences in PK, PD, or PK-PD. Moreover, it enables the evaluation of multiple or cumulative covariates that influence parameters. We considered different compartment models with first-order or zero-order elimination to determine the best fit for the KM concentration data in the population PK models. Different statistical analysis approaches can be used for the assessment of dose proportionality (linear or non-linear). In the non-compartment analysis, the t_1/2_ values were significantly different across the three doses and it is clear that the V_d_ significantly changes based on the dose. However, CL did not decrease dose proportionality in both naïve and DNP rats (Table 1). A two-compartment model with first-order absorption and elimination showed the lowest -2LL (Supplementary Table S5). Furthermore, although the 90% confidence intervals for some dose in DNP rats did not fall within dose-proportionality limits, AUC_0-∞_ were found to increase in an approximately dose-proportional manner, as shown in Supplementary Figure S4 (Darwish et al., 2006). Thus, the two-compartment model with first absorption and elimination was used for the PK/PD model. Large BSVs in vs (V_1_ or V_2_) were observed in population PK model parameters. The covariable dose effect on V_s_ may be properly adjust the nonlinearity in PK (Supplementary Table S3). Furthermore, we found that STZ-induced hyperglycemia, but not the hyperglycemia-induced low threshold, significantly influenced the PK-PD profile. Higher levels of blood glucose cause non-enzymatic glycation of many proteins, including albumin (Zini et al., 1990). Unfortunately, little is known about the protein-binding characteristics of KM. The lower V_d_ in DNP rats may be associated with conformational changes and decreased protein-binding capacity in the glycated plasma protein, necessitating further research to help address these questions accurately. Previous studies suggested that CYP3A4/5 is the major elimination pathway for KM (Zhang et al., 2013; Hu et al., 2017; Xiao et al., 2017). Further, our previous studies have reported that KM elimination occurs majorly through liver microsomes (Wei et al., 2016; Wei et al., 2017). The KM metabolic pathway in liver microsomes involves oxidization, demethylation, and dehydrogenation (Wang et al., 2019). Diabetes affects the metabolism of proteins, carbohydrates, and lipids, and may be directly or indirectly involved in drug biotransformation. Therefore, it is not surprising that the PK of drugs were affected by diabetes as reported for lidocaine, nisoldipine, and tramadol (Marques et al., 2002; Moisés et al., 2008; Godoy et al., 2019). However, the impacts of diabetes on KM PK are still unknown. In our PK/PD study, the blood glucose level prior to KM treatment significantly reduced the systemic CL (Table 2). Some studies have reported markedly reduced hepatic CYP3A4 metabolism of drugs in diabetic patients (Dostalek et al., 2011). The CL of the CYP3A4 substrates lidocaine and nisoldipine was significantly reduced in diabetic patients compared to that in the non-diabetic group (lidocaine: 5.64 vs. 10.6 ml min^−1^ kg^−1^; nisoldipine: 3.6 vs. 18.7 L h^−1^ kg^−1^) (Marques et al., 2002; Moisés et al., 2008). Taken together, the evidence implied that hyperglycemia may downregulate the enzyme activity of CYP3A4/5 responsible for the metabolism of KM. The accumulation of end products of advanced glycation and inflammation probably alters the expression and function of transporters in mice with experimentally induced diabetes (Costa et al., 2020). However, information on transporters contributing to KM elimination is limited. Wang et al. found that active transport may be one of transport processes for KM with an equivalence relation between absorptive direction and efflux direction *via* P-gp and MRP2 (Wang et al., 2017). Furthermore, it is still unclear whether KM is a substrate of any transporter. The next problem is whether these influences reflect diabetes-mediated changes in PK rather than PD.

One of the limitations of this study is that PK and PD experiments were conducted in different sets of rats. The other limitation is that the results of the covariate analysis regarding blood glucose level were limited to a maximum value of 16.6 mmol L^−1^ due to the instrument, above which the actual blood glucose values could not be obtained. These limit the direct application of the current results to determine optimal treatment strategies for clinical cases. Data from animal studies should be assessed conservatively when trying to extrapolate the changes to humans.

## Conclusion

Orally administered KM quickly induced an analgesic effect in DNP rats. However, DNP also changed the *in vivo* PK of KM. The PK-PD analysis revealed that STZ-induced hyperglycemia significantly influenced the PK and anti-allodynic actions of KM in a rat model of DNP.

## Data Availability

The original contributions presented in the study are included in the article/Supplementary Material, further inquiries can be directed to the corresponding authors.
