# Supplementary figures and images for: Streptozotocin-Induced Hyperglycemia Affects the Pharmacokinetics of Koumine and its Anti-Allodynic Action in a Rat Model of Diabetic Neuropathic Pain
Source: Front Pharmacol. 2021 May 13;12:640318. doi: 10.3389/fphar.2021.640318 (PMC8156416; doi:10.3389/fphar.2021.640318)

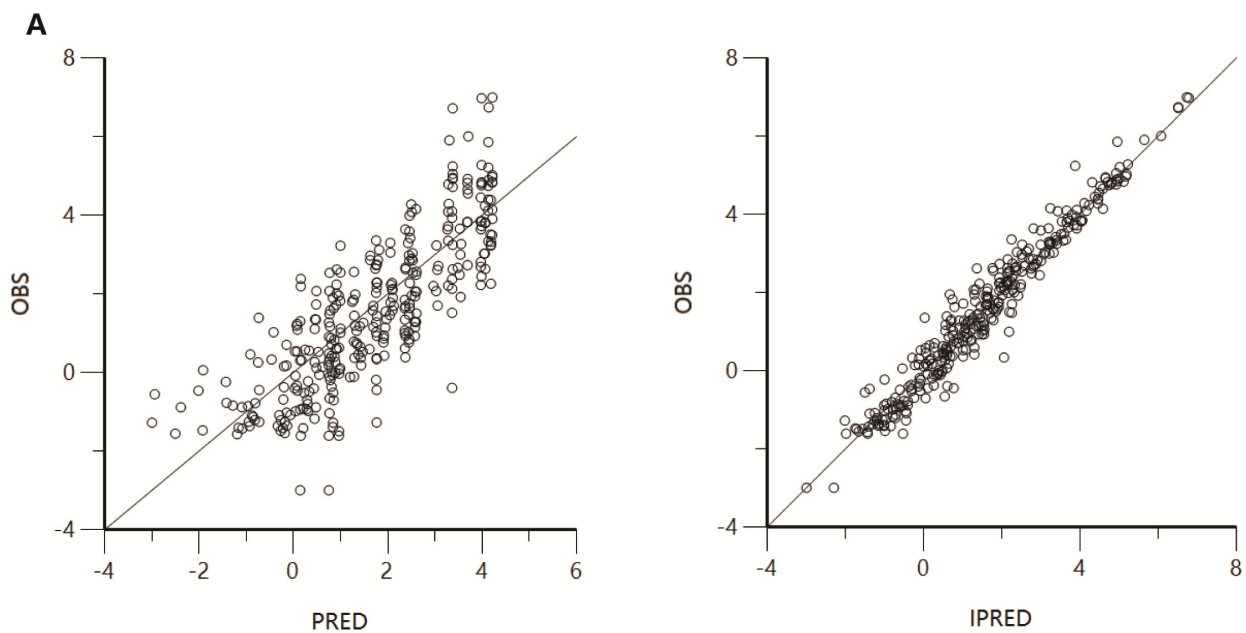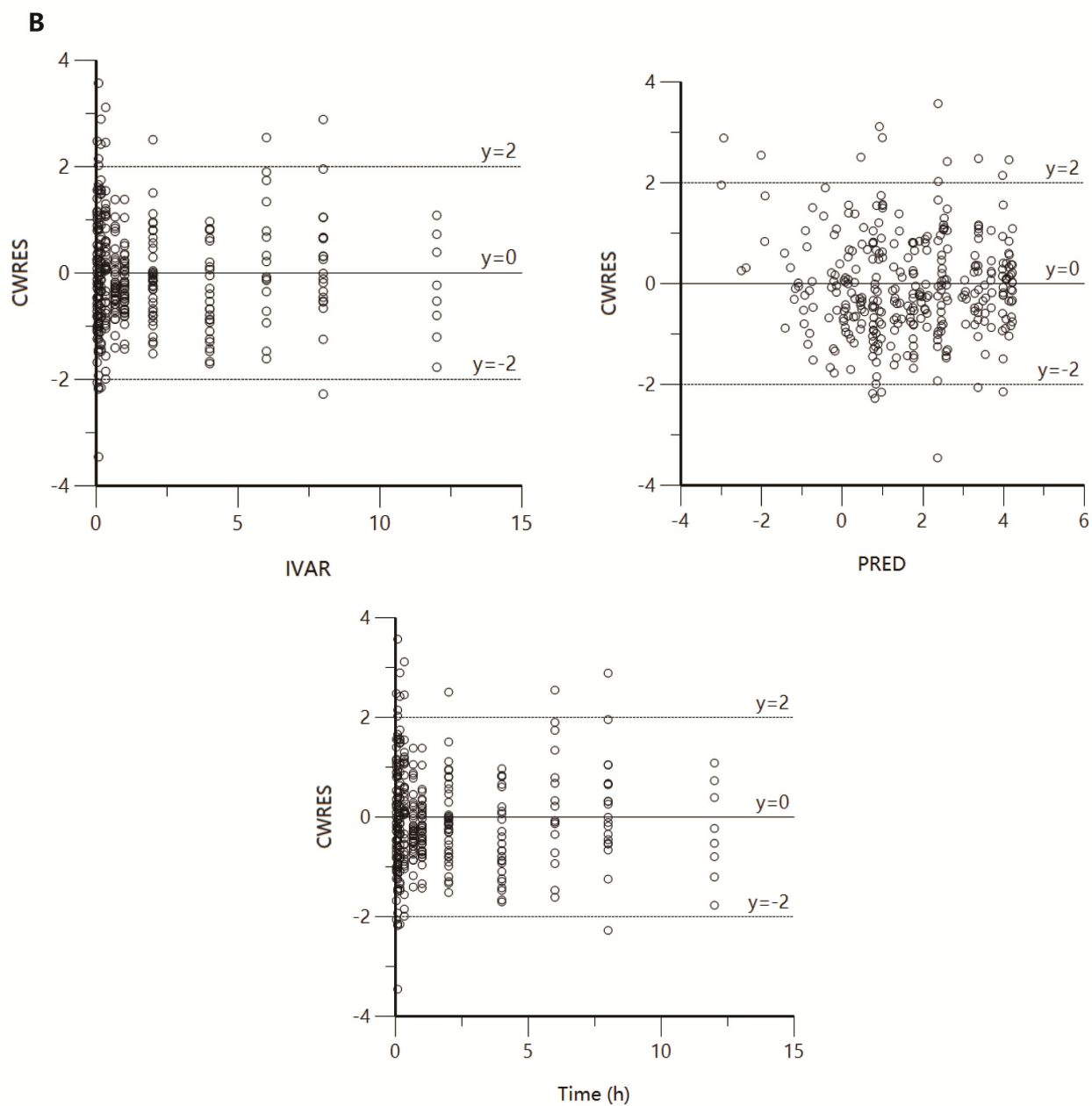

Supplement: Supplementary file 1 [file DataSheet2.PDF]

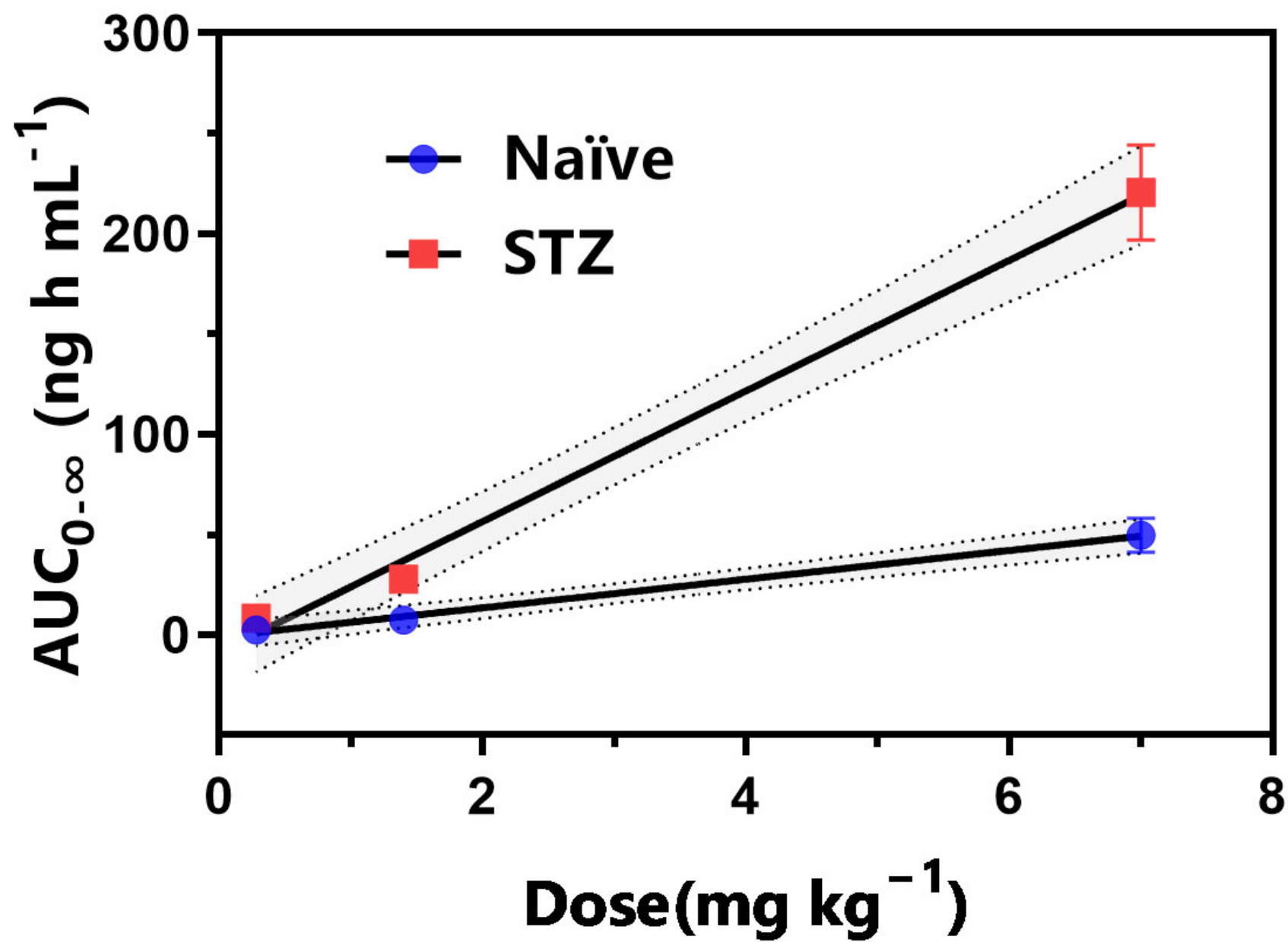

Supplement: Supplementary file 2 [file DataSheet4.PDF]

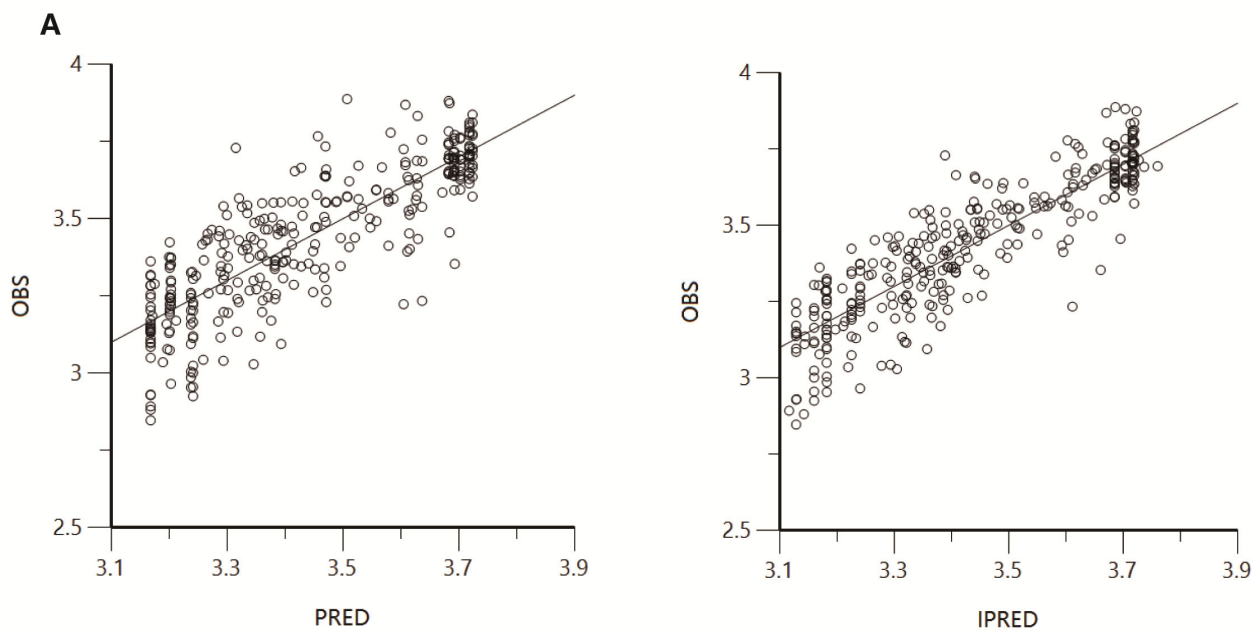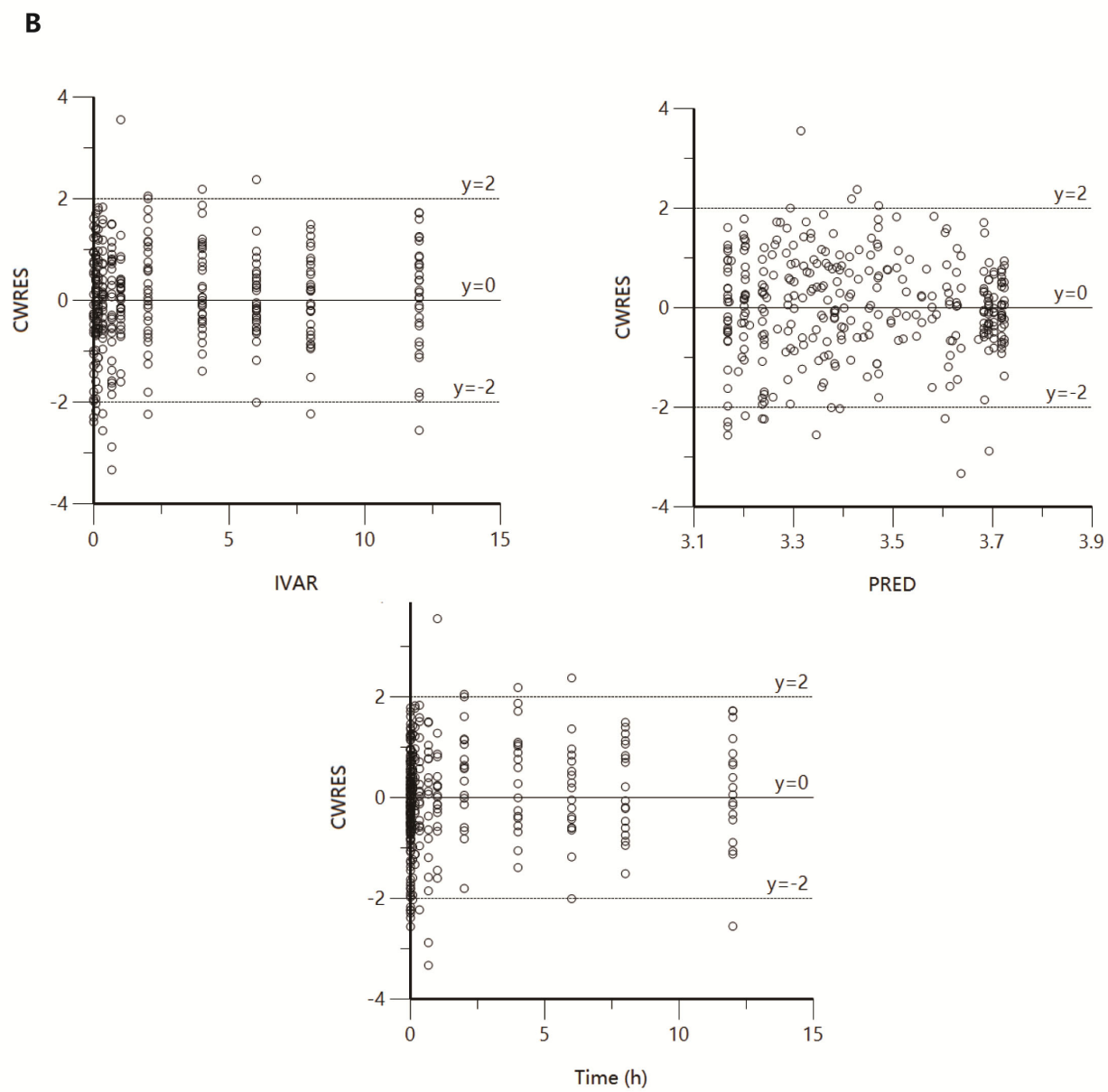

Supplement: Supplementary file 4 [file DataSheet3.PDF]

**A****KM**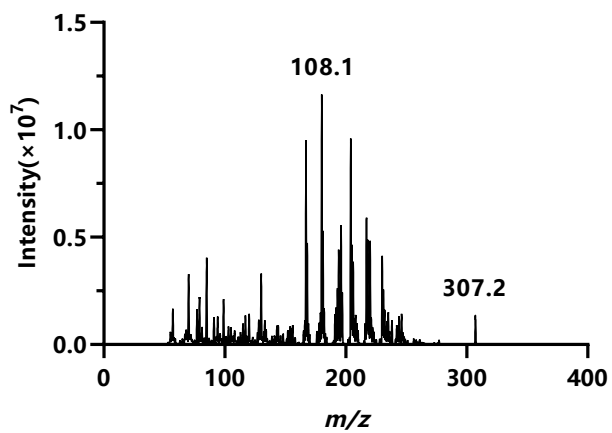**B****GM**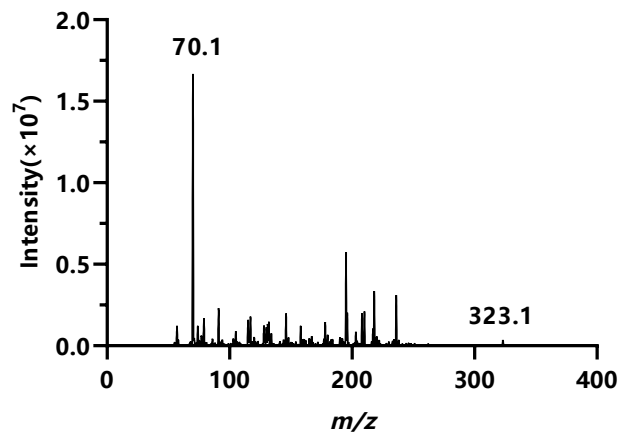**C**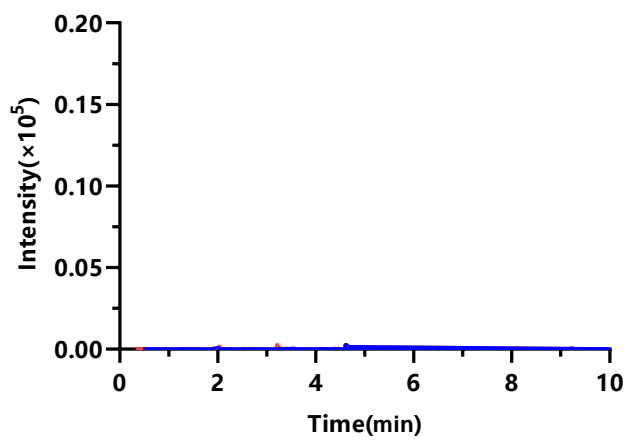**D**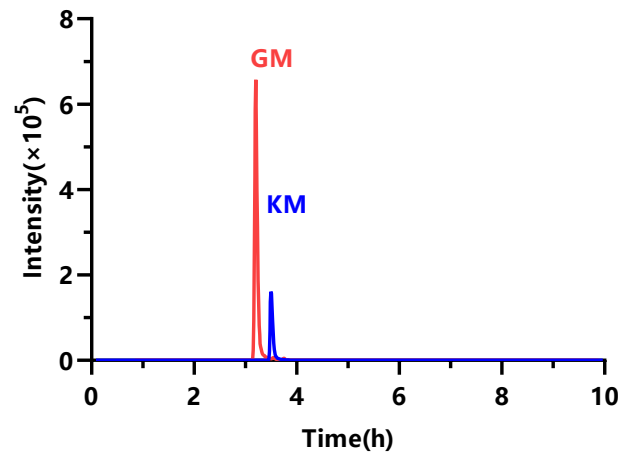**E**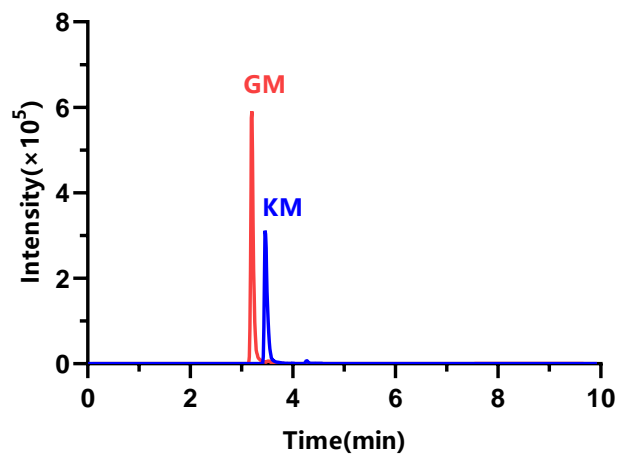**F**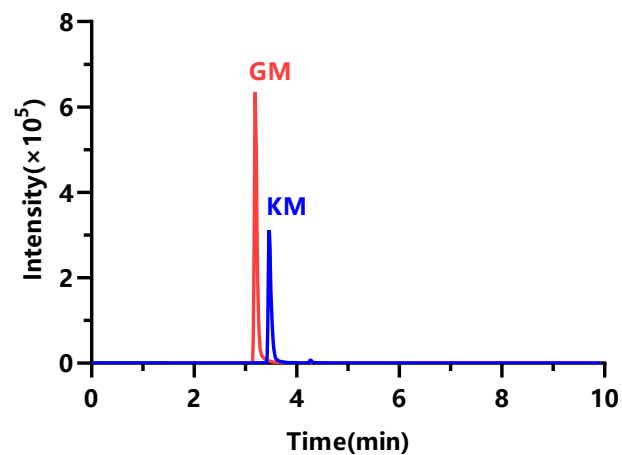

Supplement: Supplementary file 5 [file DataSheet1.PDF]
