# Supplementary material for: Streptozotocin-Induced Hyperglycemia Affects the Pharmacokinetics of Koumine and its Anti-Allodynic Action in a Rat Model of Diabetic Neuropathic Pain
Source: Front Pharmacol. 2021 May 13;12:640318. doi: 10.3389/fphar.2021.640318 (PMC8156416; doi:10.3389/fphar.2021.640318)
Supplement: Supplementary file 3 [file DataSheet6.PDF]

Table S1. The Gradient Program of KM Plasma Concentration Analysis by UPLC

Time (min)	Percentage of Methanal (%)	Percentage of Water with 0.1% Formic Acid (%)
0.0 - 1.0	20	80
1.0 - 4.0	20 → 95	80 → 5
4.0 – 5.0	95	5
5.0 – 5.01	95→20	5 → 80
5.01 – 10.0	20	80

Table S2. Precision and Accuracy of the Determination of KM Concentration in Rat Plasma (*n* = 5).

Spiked Concentration	Accuracy	Intra-Day Precision	Inter-Day Precision
(ng mL^{-1})	(%)	CV (%)	CV (%)
0.2 (LLOQ)	98.1	12.7	13.8
0.6	107.4	6.3	7.9
10	92.3	9.8	8.3
150	101.9	1.6	3.5

Table S3. Matrix Effects and Extraction Recovery of KM in Rat Plasma (*n* = 6).

Spiked Concentration ($ng\ mL^{-1}$)	Matrix Effect		Extraction Recovery	
	Mean (%)	CV (%)	Mean (%)	CV (%)
0.6	100.0	5.2	67.2	3.8
10	100.0	1.4	59.0	7.1
150	108.0	3.8	64.5	3.7

Table S4. Stability of KM in Rat Plasma under Different Conditions (*n* = 3).

Spiked Concentration ($ng\ mL^{-1}$)	3 Freeze-Thaw Cycles (%)	Short-Term Storage (%)	Long-Term Storage (%)	Post-Preparative (%)
0.6	95.8	98.3	111.0	100.4
10	109.0	86.3	94.2	109.6
150	96.9	90.7	94.0	95.5

Table S5 Comparison of the minimum value of twice the negative log likelihood (-2LL) among population pharmacokinetic models

Models	-2LL
One compartment with first-order absorption and elimination	903
One compartment with first-order absorption and zero-order elimination	1432
Two compartments with first-order absorption and elimination	821*
Two compartments with first-order absorption and zero-order elimination	823
Three compartments with first-order absorption and elimination	894
Three compartments with first-order absorption and zero-order elimination	1064

*Lowest -2LL
