# Supplementary material for: Streptozotocin-Induced Hyperglycemia Affects the Pharmacokinetics of Koumine and its Anti-Allodynic Action in a Rat Model of Diabetic Neuropathic Pain
Source: Front Pharmacol. 2021 May 13;12:640318. doi: 10.3389/fphar.2021.640318 (PMC8156416; doi:10.3389/fphar.2021.640318)
Supplement: Supplementary file 6 [file DataSheet5.pdf]

Supplement Figure Legends

Figure S1. The full-scan precursor ions and products of (A) koumine (KM) and (B) internal standard gelsemine (GM); Representative chromatograms of (C) blank rat plasma, (D) blank rat plasma spiked with KM (final concentration: 20.0 ng ml^{-1}) and GM (final concentration: 80 ng ml^{-1}), (E) Naïve rat plasma collected 0.17 h after a single oral administration of 7.0 mg kg^{-1} KM and spiked with GM (final concentration: 80 ng ml^{-1}), and (F) STZ-induced diabetic neuropathic pain rat plasma collected 0.17 h after a single oral administration of 7.0 mg kg^{-1} KM and spiked with GM (final concentration: 80 ng ml^{-1}).

Figure S2. Goodness-of-fit plots of the final population pharmacokinetics model for koumine. (A) Observed (OBS) versus population predictions (PRED, left) or individual predictions (IPRED, right) of KM concentrations. Linear regression fit is shown in black. (B) Conditional weighted residuals (CWRES) versus independent variables (IVAR, left), PRED (right), or time (bottom). Observed data are presented as black circles.

Figure S3. Goodness-of-fit plots of the final population pharmacodynamics model for koumine. (A) Observed (OBS) versus population predictions (PRED, left) or individual predictions (IPRED, right) of KM concentrations. Linear regression fit is shown in black. (B) Conditional weighted residuals (CWRES) versus independent variables (IVAR, left), PRED (right), or time (bottom). Observed data are presented as black

circles.

Figure S4. Relationship between $AUC_{0-\infty}$ and Dose. The dashed lines are the 90% confidence intervals.
